# Diversity of Group Memberships Predicts Well-Being: Cross-Sectional and Longitudinal Evidence

**DOI:** 10.1177/01461672231202278

**Published:** 2023-09-30

**Authors:** Sarah J. Charles, Clifford Stevenson, Juliet R. H. Wakefield, Emanuele Fino

**Affiliations:** 1 Nottingham Trent University, UK

**Keywords:** social identity, group-type diversity, wellbeing, loneliness, multiple group membership

## Abstract

Groups have their health and well-being impacted by satisfying their members’ needs and providing resources to help cope with threats. Multiple group memberships serve to accumulate these benefits and also provide resilience to the effects of group loss. However, the additional well-being benefits of belonging to multiple *different types of group* remain to be determined. In a preregistered cross-sectional survey in Nottingham, England (Study 1, *N* = 328), we found that group-type diversity predicted well-being and that this effect was fully serially mediated by increased creative self-efficacy, then reduced loneliness. To confirm our hypothesis in a more robust sample we conducted longitudinal analyses on the English Longitudinal Study of Aging (ELSA) dataset (Study 2, *N* = 5,838) finding that group-type diversity at time one (T1) predicted well-being at T2 (4 years later), even when accounting for wellbeing and loneliness at T1. We discuss the implications for enhancing group-based health interventions.

## Introduction

### Background

Across the health and social sciences, an increasing consensus has emerged as to the negative effects of loneliness and social isolation on mental and physical health as well as the important role-played by group memberships in improving health and providing social and psychological resilience (Holt-Lunstad et al., 2015; Holt-Lunstad & Smith, 2012). In particular, research in the Social Identity Approach to Health (SIAH) tradition has pointed to the impact of group dynamics on health, which is posited to be due to group members’ social identities (i.e., the aspect of self-identity derived from group membership) shaping their perceptions and behaviors (Jetten et al., 2009; Jetten, Haslam, & Haslam, 2012). This “Social Cure” effect has been found across family, community, sporting, therapeutic, and workplace settings as well as at population level (C. Haslam et al., 2018).

While not every group is beneficial for one’s health (see Wakefield et al., 2019), the potential health benefits of group membership have been harnessed in a series of interventions including Groups4Health (Cruwys et al., 2022; C. Haslam et al., 2016; S. A. Haslam et al., 2019) and Groups4Belonging (Dingle, Ingram, et al., 2021). These scaffold their participants’ group memberships, thereby promoting substantial and sustained health and well-being benefits. Moreover, the benefits of group memberships are cumulative, such that belonging to more groups is (on average) associated with better health and well-being (Iyer et al., 2009). This is partly due to multiple group memberships increasing one’s resilience and ensuring that losing one group membership will not overly impact health. These aspects are especially important during major life changes, which can involve both stress and group loss (S. A. Haslam et al., 2019; C. Haslam et al., 2021).

Missing from this line of research is the consideration of how belonging to multiple different *types* of groups (i.e., being a member of one sports group and one religious group, compared with two sports groups) can have added health and well-being benefits. At the individual level, there is a wealth of evidence suggesting that exposure to diverse experiences can have psychological, social, and health benefits (Gocłowska et al., 2018). For example, biculturalism has been shown to have the potential to positively impact individuals’ health and well-being, in part through their access to two sets of identity-related resources (Repke & Benet-Martínez, 2019). Belonging to multiple groups has also been shown to increase creativity (Steffens, Jetten, et al., 2016). Moreover, at group level, groups with more diverse memberships tend to be more creative and resilient (e.g., Han et al., 2014). Yet the added benefits of group-type diversity and the specific benefits that this may have for members’ well-being has been largely overlooked.

The present study aims to redress this imbalance by considering how and why belonging to different types of groups may accrue psychological benefits, which may in turn benefit members’ health and well-being. We then directly test our hypothesis that group-type diversity predicts improved well-being in community and population samples, before considering the processes through which this relationship may occur.

### The Social Identity Approach to Health

The SIAH (C. Haslam et al., 2018) focuses on the different ways in which group memberships impact health and wellbeing (well-being is defined by the WHO as encompassing “quality of life and the ability of people . . . to contribute to the world with a sense of meaning and purpose” (World Health Organization, 2021, p. 10]). At its core is the understanding that groups shape both their members’ perceptions and their individual and collective responses to their environments. Building on the appraisal model of stress developed by Lazarus and Folkman (1984), the SIAH proposes that group identity impacts upon both the primary and the secondary stress appraisal processes of their members. In terms of primary appraisal, group identity serves to shape group members’ perceptions of their environment, dictating whether a specific stimulus is likely to be perceived as threatening or benign. For example, pilgrims at a religious festival in Northern India were found to experience the loud cacophonic noise of the gathering as peaceful and blissful, but only insofar as it accorded with their religious identity (Shankar et al., 2013). Similarly, Jones and Jetten (2011) found that men performed better on a cold-pressor task if they were primed with masculine norms of high pain tolerance.

In terms of secondary appraisal, groups impact upon the extent to which their members perceive themselves as being able to cope with threats. Groups can provide social and material resources to enable their members to cope more effectively and can come together to collectively respond to challenges. For example, at the aforementioned Indian festival, the understanding that other attendees would provide assistance enabled pilgrims to endure extremely cold weather (Pandey et al., 2014). Likewise, bomb-disposal experts were found to experience the challenges of their work as no more stressful than those facing bartenders, but this depended upon the experts perceiving their colleagues as an important source of support thereby making their challenging job more manageable (S. A. Haslam et al., 2005).

These appraisal processes typically operate in tandem, such that the effects of group membership on perceptions of potentially stressful stimuli occur in parallel with the impact of receiving support from others in the group. For example, a recent study exploring the relationship between neighborhood identification, loneliness, and well-being showed that neighborhood identification was a direct negative predictor of loneliness but that it also was an indirect predictor via increased social support (McNamara et al., 2021). In other words, neighborhood identification both made people feel less lonely *and* gave them access to social resources which tempered their experience of loneliness.

Of course, groups do not inevitably benefit their members’ health, and there are many examples of negative identity dynamics whereby group members are denied support and left vulnerable (Kellezi & Reicher, 2011). Similarly, if groups lack resources, then the degree of benefit to be gained from membership can be limited. For example, in an economically deprived neighborhood, the availability of family support was found to moderate the benefit of family identification upon financial stress: If families lacked the wherewithal to help, then they were of limited assistance (Stevenson et al., 2022). However, in the main, individual groups do serve as a pool of social and psychological resources for their members.

### Multiple Group Memberships

If individual groups can positively impact health and well-being, then belonging to multiple groups can multiply these benefits (Iyer et al., 2009). This has been found to occur through two broad routes. First, the effects of group memberships can be cumulative such that belonging to multiple groups simply affords access to more social and psychological support (Jones & Jetten, 2011). For individuals facing social or economic challenges, having multiple sources of support will be of greater benefit than relying on a single (and possibly finite) set of resources (Stevenson et al., 2022). As Cruwys et al. (2013) demonstrate using the English Longitudinal Study of Aging, the number of groups to which an individual belongs predicts lower depression and increased resilience to depression relapse over time.

Second, belonging to multiple groups provides an individual with resilience to life changes (Cruwys et al., 2013). As posited by the Social Identity Model of Identity Change (SIMIC), major life transitions such as illness, retiring, bereavement, or moving home are often accompanied by substantial loss of social connections, including group memberships, which can have a serious effect on health and wellbeing (C. Haslam et al., 2021). The model proposes that having multiple group memberships can offset this by providing some degree of identity continuity as well as continued support. For example, a nationally representative sample of older adults indicated that losing group memberships can lead to a sixfold increase in mortality likelihood in the first 6 years of retirement (Steffens, Cruwys, et al., 2016), while multiple group memberships have been shown to operate as a resilience factor during stroke recovery, as the maintenance of these groups is associated with better well-being post-stroke (C. Haslam et al., 2008).

Once more though, multiple group memberships are not always beneficial for health and well-being. For example, problems may arise if the groups in question are incompatible (i.e., they possess conflicting values; Sønderlund et al., 2017). In such cases, the individual may experience discord in their sense of self as well as practical difficulties in maintaining incompatible memberships (Brook et al., 2008). In the example of working-class students moving to university, a clash between the values of their backgrounds and the largely middle-class ethos of universities poses a challenge for those wishing to retain some degree of identity continuity (Iyer et al., 2009). Conflicting group memberships have also been shown to predict reductions in well-being for individuals whose sense of self is fixed rather than flexible (Rabinovich & Morton, 2016), and for bicultural youth (Rahim et al., 2021). On the contrary, incompatibility between an addict or eating disorder identity and a family or friendship group identity may assist successful transition away from problematic groups, thus providing an exit while scaffolding rehabilitation (Best et al., 2016).

Despite this appreciation of the effects of the number of social relationships on health and well-being, the SIMIC does not consider the potential benefits of belonging to different *types* of groups. In principle, belonging to a diversity of group types should have added benefits for resilience to challenge and change due to different groups providing their members with access to different types of support. Specifically, belonging to a greater variety of groups should meet a wider range of individual members’ needs by increasing the range of resources and strategies that can be brought to bear on coping with potential threats and challenges. For an understanding of what this might look like, we need to turn to other research on the psychological effects of diversity.

### The Potential Benefits of Diversity

A wide range of literature attests to the psychological and health benefits of diversification. At both the individual and group level, exposure to the diversity of experiences and perspectives is linked to enhanced creativity and creative self-efficacy (e.g., Gocłowska et al., 2018; Hundschell et al., 2022), and in turn, creative self-efficacy is a predictor of health and well-being (e.g., Al-Dhaimat et al., 2020; Mangion & Konietzny, 2022). However, evidence of relationships between diversity, creativity, and well-being is stronger in research that focuses on the individual (Forgeard & Benson, 2019), whereas this relationship has been under-explored in research that focuses on the group. We consider both contexts below.

### Individual Diversity and Well-Being

Diversity has been shown to have multiple psychological and well-being benefits at the level of the individual. For example, exposure to diversifying experiences (experiences that challenge existing schemas or stereotypes) has been shown to increase cognitive flexibility, problem-solving, self-esteem, and self-efficacy (Crisp & Turner, 2011). Indeed, the Diversifying Experiences Model (Gocłowska et al., 2018) specifies the condition under which diversifying experiences have the aforementioned positive benefits: If individuals have the resources to cope with diversifying experiences, these experiences will more likely result in enhanced creativity.

In turn, creativity impacts positively on well-being through various pathways. Creative self-efficacy (the belief that one is creative) has been found to predict lower stress among gifted students (Al-Dhaimat et al., 2020) and higher well-being among primary school students (Mangion & Konietzny, 2022) as well as to mediate the relationship between individual personality differences and wellbeing among undergraduate students (Fino & Sun, 2022). Similarly, creative self-efficacy has been shown to mediate the relationship between creative adaptability and well-being during challenges such as COVID-19 (Orkibi, 2021), a pattern that holds across international contexts (Orkibi et al., 2021). In effect, creativity typically has its well-being benefits through an enhanced belief in one’s creative abilities.

### Group Diversity and Well-Being

At the group level, the diversity of members within groups has been shown to have benefits for group problem-solving and innovation. More diverse teams have been found to be more creative due to increased bridging and bonding social capital (Han et al., 2014). Elsewhere the diversity of social ties in a workgroup has been found to predict creativity through enhanced creative self-efficacy (Gong et al., 2020), while participation in extra-curricular activity groups was found to have a positive relationship with mental health (lower depression and anxiety, and higher well-being), in part through creative self-efficacy (Forgeard & Benson, 2019).

In terms of belonging to multiple different types of groups, there is substantial evidence from the study of biculturalism that, under favorable circumstances, membership of two or more cultural backgrounds enhances creativity and integrative complexity (Tadmor et al., 2009), and positively impacts well-being. When bicultural individuals can maintain both identities harmoniously, they can benefit from the social and cultural resources of both heritages, using these to more flexibly and creatively address challenges, including challenges of acculturation (maintenance of one’s own ethnic identity and adoption of the host identity). In turn, successful acculturation has been found to positively predict lower acculturative stress and better health and well-being (Gocłowska & Crisp, 2014).

Social Identity Approach researchers have also found relationships between group memberships and creativity. In terms of single groups, S. A. Haslam and colleagues (2013) theorized that group membership provide the motivation for creativity to address shared problems, align with group norms of creativity, or, in the case of new groups, to inductively develop new behavioral norms. Groups also provide the framework within which creativity is valued or rejected, with high-identifying students (compared with low-identifying students) rating the novel ideas of a fellow student more positively (Adarves-Yorno et al., 2006). Researchers have also investigated the impact of multiple group memberships on creativity. Steffens, Gocłowska, et al. (2016) predicted that, insofar as different identities provide different ways of viewing the world, belonging to a greater number of groups should provide more insights and solutions to potential challenges. In a series of studies, they showed that belonging to multiple groups does demonstrably predict increased “idea originality” (operationalised via generating names for a commercial product or uses for a brick) through increased cognitive flexibility. This effect was shown to occur independent of task perseverance, self-affirmation, or novelty seeking.

However, we note some limitations of this body of work. This research remains discrete from the broader SIAH work on groups and health, and so the well-being implications of the creativity-enhancing effects of groups remain unexplored. The broader research on creativity indicates some possible ways in which increased creativity should benefit individuals’ health and well-being. However, the degree to which this operates for group memberships, and the pathways through which this might operate, remain unexplored. Moreover, the specific benefits of belonging to multiple different group types (rather than many of the same type of group) are yet to be investigated. Insofar as we would expect insights and solutions to be afforded by different group types, the extent of their diversity should be critical for members’ health and well-being. In other words, we would expect creativity to be an emergent property of belonging to multiple different types of groups, *over and above* belonging to more of the same type of group.

### Diverse Group Memberships, Creative Self-Efficacy, and Well-Being

One promising explanatory mechanism for the relationship between membership of a diversity of group types and well-being is self-efficacy (Bandura et al., 1999), and more specifically, creative self-efficacy beliefs. Creative self-efficacy has been defined as a system of beliefs in one’s own ability to innovatively perform specific tasks (Beghetto & Karwowski, 2017), ingeniously solve problems (Fino & Sun, 2022), generate creative outcomes (Tierney & Farmer, 2002, p. 1138), and efficiently rise to challenging or stressful situations (Shaw et al., 2021). These beliefs have been conceptualized as being future-oriented, task-specific, and dynamic (Beghetto & Karwowski, 2017), in that they enable individuals to flexibly manage and adapt cognitive resources to generate highly functional responses to stressful situations and difficult tasks (Choi, 2004), which predicts “whether a person will engage with (or avoid) a particular performance opportunity [. . .], sustain effort [. . .], perform at a particular level of creative achievement [. . .], and ultimately judge themselves as creative in various performance domains” (Beghetto & Karwowski, 2017, p. 4).

Given that we know that the benefits of groups for health rests on their ability to provide problem-solving resources, we propose that creative self-efficacy may therefore provide a link between diverse group types and well-being: members of diverse group types are more likely to feel more resilient to future challenges. Moreover, we suggest that a further pathway from group type diversity and creativity to well-being may be through loneliness reduction. While some social identities have been found to reduce loneliness among members (e.g., Ingram et al., 2020; McNamara et al., 2021), diverse group types may additionally help reduce loneliness through increasing creative self-efficacy. Loneliness is associated with lack of cognitive flexibility (Akdeniz & Gültekin Ahçı, 2023) and attention bias toward negative social stimuli and social threats (Cacioppo & Hawkley, 2009), which becomes a self-fulfilling prophecy. Increased creative self-efficacy, and its accompanying cognitive flexibility, could potentially serve to offset these effects by enabling cognitive reappraisal of one’s social situation, thereby interrupting the self-perpetuating cycle of loneliness.

### Background for Hypotheses

Building upon the previous research into the different ways in which single and multiple group memberships benefit health and wellbeing, we suggest that examining the diversity of group types of which individuals are members is worthy of attention in its own right. We propose that diversity of group types is likely to predict enhanced creative self-efficacy, and that this, in turn, will predict better wellbeing. Furthermore, we suggest that one way in which creative self-efficacy may improve wellbeing is by reducing loneliness.

## Study 1

### Rationale and Context

Study 1 was a pilot study pre-registered via OSF (https://tinyurl.com/GroupDiversity) designed to explore the relationship between group-type diversity and well-being. These data were collected in the Ashfield district in the county of Nottinghamshire, which is in the East Midlands of England. This district was selected because of its relatively low socioeconomic status and the associated well-being–related challenges its residents have experienced both before and after the COVID-19 pandemic.

We felt it was important to use two of the most frequently used and well-validated measures of well-being: the WHO5 general well-being measure (World Health Organization, 1998) and the UCLA Loneliness Scale (Hughes et al., 2004). In doing so, we hope that the current study will apply to those researching biopsychosocial sociopsychological approaches to health, irrespective of their more specific outcome foci.

### Pre-Registered Hypotheses

Given the theoretical approach we are taking (SIAH), with a specific interest in the role of group-type diversity, we pre-registered the following specific hypotheses that we wished to test in this pilot study:

1. Greater group-type diversity would be significantly associated with lower feelings of loneliness, as measured by the UCLA Loneliness Scale.
2. Greater group-type diversity would be significantly associated with a greater level of general wellbeing, as measured by the five-item World Health Organization Wellbeing Index (WHO-5) scale.

Beyond these pre-registered hypotheses, further exploratory analyses, such as path analyses, were conducted to explore whether the relationship between group-type diversity and well-being is mediated by creative self-efficacy.

## Method

OSF Repository: https://osf.io/36bvz/?view_only=0c976b8fd5794cf1babf8351528a5423

### Participants and Procedure

This study was pre-registered after data collection had taken place, but before data analysis. In total, 29,835 invites to take part in the survey were sent out to Ashfield residents via Royal Mail (all Ashfield postcodes were covered by this mail drop). The invite contained a link to an online survey (Qualtrics, 2019) that took approximately 15 min to complete. Three-hundred and eighty-eight residents chose to participate. Of these, 254 identified as women, 131 identified as men, and 3 identified as non-binary. Of the 388 participants, 328 (84.5%) completed enough of the questionnaire to provide data for both outcome variables of interest in our preregistered hypotheses. Given our knowledge of our sample size, a sensitivity power analysis was conducted to determine the smallest effect size that could be reliably detected.

We conducted sensitivity power analyses for our two main hypotheses. For the rationale behind the choice of parameters, please see the online Supplemental Material. For the first hypothesis, using the pwr.r.test() function from the pwr package (v.1.3.0) in R (Champely et al., 2020), the following parameters were used: *n* = 328, sig.level = 0.1, power = 0.8, and alternative = “less.” In this case, an effect size of *r* = -0.12 (2.d.p.) could be reliably detected. If the effect size was lower than this (in magnitude), we would consider it too small to be of interest.

For the second hypothesis, using the pwr.r.test() function, the following parameters were used: *n* = 328, sig.level = 0.1, power = 0.8, and alternative = “greater.” In this case, an effect size of *r* = .12 (2.d.p.) could be reliably detected. If the effect size is lower than this (in magnitude), we will consider it too small to be of interest, that is, our smallest effect size of interest for our pre-registered hypotheses is *r* =|.12.| If the effect size we detected was larger, in magnitude, than 0.12 then it would be worthy of note.

### Measures

Some measures were included in the survey for exploration in a separate piece of research, so are not reported here. A list of all manipulations, measures, and exclusions is contained in the Supplemental Material document on the aforementioned OSF project. For multi-item measures, the internal consistency is given in both Cronbach’s alpha (α) and McDonald’s total omega (ω).

#### Group Type Diversity

Participants were asked a set of ten questions, with a binary yes/no response. Nine of the questions were structured as “Do you belong to one or more [GROUP-TYPE] groups?” Each of these nine questions replaced [GROUP-TYPE] with a specific option: (sport/ tenant/ political/ religious/ charitable/ educational/ social/ support/ other). The 10^th^ question, “Do you belong to no groups?,” was provided, also with a binary response, as an attention check question.

A similar question appears in the English Longitudinal Study of Aging (ELSA; Marmot, 2020; Marmot et al., 2003), which asks about eight of the same group types (all of the above, excluding “Support”). We added the “Support” group type based on evidence from more recent research that suggests it is its own type of group (see Sani et al., 2015).

Previous uses of this scale (e.g., Cruwys et al., 2013) have simply totaled the number of positive responses to give an indication of group memberships. However, we elected to score this variable differently to capture group type *diversity* rather than simply number of groups. If participants responded that they were part of either no groups or only a single type of group, they would be scored as having no (zero) group-type diversity. Each group type after the first would then be added to their group-type diversity score (i.e., being a member of four group types would give a group-type diversity score of three).

#### Creative Self-Efficacy

To measure creative self-efficacy, we used the three-item Creative Self-Efficacy Instrument (Tierney & Farmer, 2002, 2011) Each item (e.g., “I have confidence in my ability to solve problems creatively”) was rated on a 1 to 7 scale (strongly disagree-strongly agree). The higher the score, the greater the participant’s level of creative self-efficacy. This measure had an internal consistency of *α* = .86, *ω* = .86.

#### Loneliness

Loneliness was assessed with the three-item The UCLA-3 (Hughes et al., 2004; Russell, 1996). Participants rated their agreement with each item (e.g., “How often do you lack companionship?”) on a 1-3 scale (hardly ever/some of the time/often). A higher score meant that they were more lonely. The internal consistency in this study was *α* = .88, *ω* = .89.

#### Wellbeing

The WHO-5 (World Health Organization, 1998) was used as a measure of general well-being. The measure asks participants to rate how well the five statements (e.g., “I have felt cheerful and in good spirits”) relate to them *over the last 14 days* using a 0 to 5 scale (at no time-all of the time). As per instructions, participants’ scores were summed then multiplied by 4 to create a 0-100 scale. A higher score meant that they had a greater level of well-being. The internal consistency was *α* = .90, *ω* = .93.

As with our choice of alpha in the power analysis, we have used an alpha (cut-off) for a significance of 0.1. See the Supplementary Materials for the rationale for the choice of alpha value.^1^

## Results

The full analysis output has been made available via an R Script file on the aforementioned OSF project. Due to the nature of our correction method, whether an effect would remain significant after correction is only known after all tests were conducted. As such, we proceeded with further testing based on estimates that the effect might be strong enough to survive correction. Because of this, both the original and the corrected *p*-values have been provided.

### Descriptive Statistics

The most common type of group that participants identified as being a member of was sports groups (*n* = 92) followed by political (*n* = 40) and Religious (*n* = 38) or other groups (*n* = 38). Table 1 shows the descriptive statistics for the four variables in this study, and Table 2 shows the correlation between measures of interest. Only pre-registered correlations were tested using inferential testing.

**Table 1:** Descriptive Statistics for Study 1 Variables.

Variables	*N*	*M*	*SD*	Range
Group Type Diversity	388	0.79	1.06	0–5
Well-being	329	49.86	23.31	0–100
Loneliness	332	1.73	0.65	1–3
Creative Self-Efficacy	331	4.99	1.28	1–7

**Table 2.** Correlation Between the Variables in Study 1 (Pearson’s r).

Variables	Group-type diversity	Well-being	Loneliness
Group-type diversity			
Well-being	.126		
Loneliness	–.056	–.557	
Creative Self-Efficacy	.183	.242	–.189

### Pre-Registered Analyses

The first pre-registered hypothesis was that group type diversity would be significantly negatively associated with loneliness. Only participants who responded to questions on both measures were included in the analysis. A one-way correlation revealed a non-significant effect, even before correcting for multiple tests, of group type diversity on loneliness, *t*(330) = −.487, *r* = −.03 [CIs: −1.00, .06], *p* = .313.

The second pre-registered hypothesis was that group type diversity would be significantly positively associated with well-being. Only participants who responded to questions on both measures were included in the analysis. A one-way correlation revealed a significant positive relationship, *t*(327) = 1.68, *r* = .09 [CIs: .00, 1.00], *p* = .047.

### Exploratory Mediation Models

Given that there was no significant relationship between group type diversity and loneliness, no further models were conducted with loneliness as an outcome measure. We only ran follow-up models to explore potential mediators of the effect between group type diversity and well-being. Only participants who answered questions relating to all measures of interest were included in the analysis (*N* = 321).

**Figure 1.**
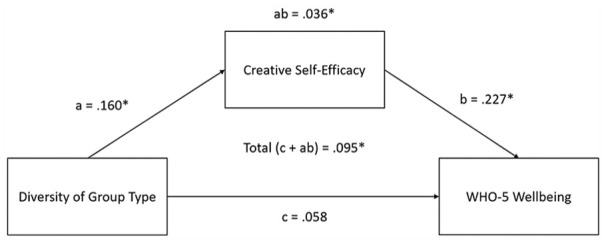
The First Mediation Model, Assessing the Mediating Effect of Creative Self-Efficacy on the Relationship Between Group Type Diversity and Well-Being. *Note.* The direct effect that was shown to be significant when only testing for the direct effect is accounted for by the significant indirect effect (ab). The standardized estimates for each effect is shown. Effects marked with an asterisk (*) were significant.

In the first model, we sought to assess whether creative self-efficacy mediated the effect of group-type diversity on well-being. Analyses were conducted in the R programming language (R Core Team, 2020) using the sem() function from the lavaan package (Rosseel, 2012, v. 0.6.14). The full outputs providing all raw and standardized estimates of the mediation models are provided in Supplemental Material on the OSF (under the heading ‘Study 1 Full Model Outputs’; https://osf.io/498rp). A mediation analysis showed that the relationship between group-type diversity and wellbeing was fully mediated by creative self-efficacy (see Figure 1). Both the indirect effect (β = .036, *Z* = 2.42, *p* = .016) and total effect (β = .086, *Z* = 1.72, *p* = .086) were statistically significant after correcting for multiple tests, but not the direct effect (β = .058, *Z* = 1.06, *p* = .288).

**Figure 2.**
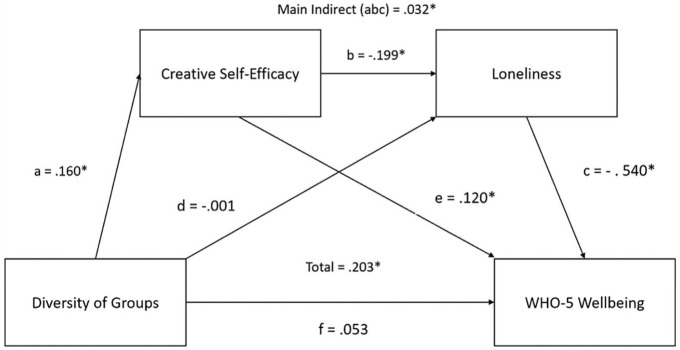
The Second Mediation Model, Assessing the Serial Mediation Effect of Creative Self-Efficacy and Loneliness on the Relationship Between Group Type Diversity and Wellbeing. *Note.* The standardized estimates for each effect is shown. Effects marked with an asterisk (*) were significant. WHO-5 = five-item World Health Organization Wellbeing Index.

As a follow-up to this mediation analysis, we tested whether a serial mediation model was a good fit for the data, where loneliness might further mediate the relationship between creative self-efficacy and well-being. This was conducted because we thought that creative self-efficacy may predict changes in how one cognitively appraises loneliness, whereby those who identify as being part of a greater diversity of groups would have higher creative self-efficacy, which would predict lower self-reported loneliness, which would itself predict greater well-being. A serial mediation analysis showed that the proposed effect of group type diversity on well-being was fully serially mediated by creative self-efficacy and loneliness, full indirect (abc) effect: β = .017, *Z* = 2.26, *p* = .024. The total effect (abc+ae+dc+f) was also significant (β = .095, *Z* = 1.72, *p* = .086). The indirect pathway fully accounted for the overall impact of group type diversity on wellbeing, with the direct effect being non-significant (β = .053, *Z* = 1.15, *p* = .250; see Figure 2).

**Figure 3.**
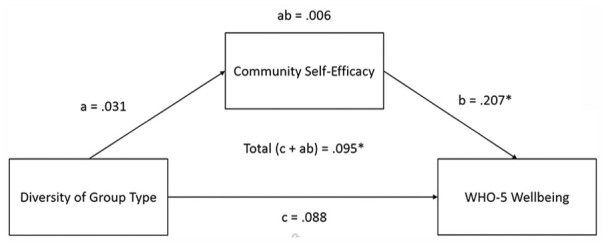
The Mediation Model Based on Häusser et al. (2020), Assessing the Mediating Effect of Collective (Community) Self-Efficacy on the Relationship Between Group Type Diversity and Wellbeing. *Note.* The standardized estimates for each effect is shown. Effects marked with an asterisk (*) were significant. WHO-5 = five-item World Health Organization Wellbeing Index.

As well as these models, we assessed the model inspired by Häusser et al. (2020), who proposed that group identification can predict greater levels of well-being via collective (rather than creative) self-efficacy. As shown in Figure 3, collective self-efficacy (in this case community self-efficacy) does not mediate the relationship between group-type diversity and wellbeing, as the indirect effect was non-significant (β = .006, *Z* = 0.55, *p* = .585), although the direct effect was no longer significant (β = .088, *Z* = 1.63, *p* = .103).

## Study 1 Discussion

Supporting pre-registered Hypothesis 2, we found that group-type diversity positively predicted well-being. It did not predict loneliness, however (thus not supporting pre-registered Hypothesis 1). Follow-up mediation analysis showed that the relationship between group-type diversity and well-being was serially mediated by increased creative self-efficacy and reduced loneliness. All results remained significant after controlling for social support, suggesting that the mechanism of social support offered by group membership is not what contributes to the relationship between diversity of group type and well-being. These findings provide some initial support for our predictions regarding the role of group-type diversity in predicting health and well-being, as well as pointing to potential mechanisms through which this relationship may occur. Study 2 was designed to corroborate these findings.

## Study 2

We felt it was important to corroborate Study 1’s findings with a longitudinal dataset covering a wider geographical area. We selected the ELSA dataset. This dataset was chosen because the authors were aware that it contained a specific question about membership of different types of groups (i.e., a measure of group type diversity).

Here, we sought to test whether the group-type diversity at Time 1 (T1) would predict well-being at Time 2 (T2) while accounting for both well-being and loneliness at T1. We did this because we wanted to build on the findings of Study 1 to help account for the role of social connections, in the form of group-type diversity, in predicting long-term well-being. The reason to assess the role of social connection on long-term well-being is that it has been shown to predict long-term well-being and loneliness (Bu et al., 2020; Cacioppo et al., 2006, 2010; Holt-Lunstad et al., 2015).

## Method

For a full description of the methods used to collect the data for the ELSA, you can see the original study published by Marmot and colleagues (Marmot et al., 2003).

### Waves Included

There is a gap of about 2 years between each ELSA wave. Due to the survey’s design, the questions asked differed slightly at each wave. As such, we had to ensure we chose waves that asked the same question(s) for each of our variables of interest, to ensure appropriate tracking over time. We chose Wave 1 for the first time point as this was the initial point of data collection, which has all the participants’ base-level information. The variables of interest were (a) group-type diversity, (b) well-being, and (c) loneliness. We had also hoped that there might be some questions about creative self-efficacy, to replicate the type of analysis conducted in Study 1, but the dataset did not contain such a measure, even in an analogous form. Wave 2 did not include the same question for loneliness as in Wave 1, but Wave 3 did contain all the same measures of interest that appeared in Wave 1. Waves 4+ did not contain the same measure of loneliness as in Waves 1 and 3, and so only Waves 1 (hereafter T1) and 3 (hereafter T2) were included in the analysis.

### Participants

A total of 7,976 participants (56.8% women) completed the ELSA at both T1 and T2. For this analysis, only participants who responded to all three measures of interest at both time points were included. After exclusion for non-response, 5,838 participants (56.0% women) were included in the analysis.

Pan and colleagues (Pan et al., 2018) produced a series of simulations suggesting that, for longitudinal structural equational models, sample sizes in the mid-100s (e.g., 300–700) are appropriate, even assuming a very low effect size and a very high intraclass correlation with only two timepoints (see Online Supplemental Material, under the section “Supplement to Method of Study 2,” for more information). Given this study has 5,838 participants, it should be appropriately powered.

### Measures

The ELSA did not have identical measures for loneliness or wellbeing, but they did have analogous measures of loneliness and wellbeing, which are outlined in more detail, below.

#### Group-Type Diversity

Participants were asked a set of eight questions, with a binary yes/no response. As with the question in Study 1, the questions were all structured as “Do you belong to one or more [GROUP-TYPE] group?” The questions replaced the [GROUP-TYPE] for a specific option that were the same as those from Study 1, only without the “support” option. The group type with the largest affiliation was “other” (*N* = 1,817), with the second highest being “sports” (N = 1,691), and “religious” (N = 1,519).

#### Wellbeing

The ELSA does not use a specific measure of well-being. Instead, they ask a set of questions about psychological health (questions labeled as psced*, where “*” is replaced by letters a-h). This is a set of eight binary response (yes/no) questions, each phrased as “Now think about the past week and the feelings you have experienced. Please tell me if each of the following was true for you much of the time during the past week. Much of the time during the past week . . .” An example of these include: “. . . you felt depressed?” “. . . you felt that everything you did was an effort?” “. . . your sleep was restless?” and “you were happy?” (reversed). A yes answer would be scored a 1 and a no answer would be scored a 2. The positively-worded questions are reversed, such that the higher value is indicative of positive wellbeing. At T1, the internal consistency of this measure was *α* = .81, *ω* = .85, and at T2, it was *α* = .82, *ω* = .86.

#### Loneliness

The measure of loneliness measure is a single question asking: “Which statement do you agree with more strongly?,” with a seven-point Likert-type scale provided with two prompts: “I have never felt lonely living in this area,” which scores 1, and “I often feel lonely living in this area,” which scores 7.

### Procedure

The ELSA was accessed via the UK Data Service (UKDS; https://ukdataservice.ac.uk/). When access was gained to the data, appropriate analog variables to those used in Study 1 were identified by the author (Author 1). The most appropriate analogue variables were listed in an excel spreadsheet which provided a comparison of the content of each of the variables. Author (Author 2) then double-checked these variables and an agreement between these two authors was reached as to which variables from the ELSA dataset to include.

Data analysis was conducted using the lavaan package (Rosseel, 2012) in *R* (lavaan v. 0.6.14). The analysis conducted leads to the creation of multiple *p*-values due to multiple *Z*-tests being conducted. However, as the tests are not disjunction tests (see Rubin, 2021) there is not a need to correct for multiple tests (see also Armstrong, 2014). However, as this study was conducted as a follow-up to seek to confirm the effects found in Study 1, the alpha value was lowered to 0.01, to reduce the likelihood of a Type I error, which is important for confirmatory analyses.

## Results

**Figure 4.**
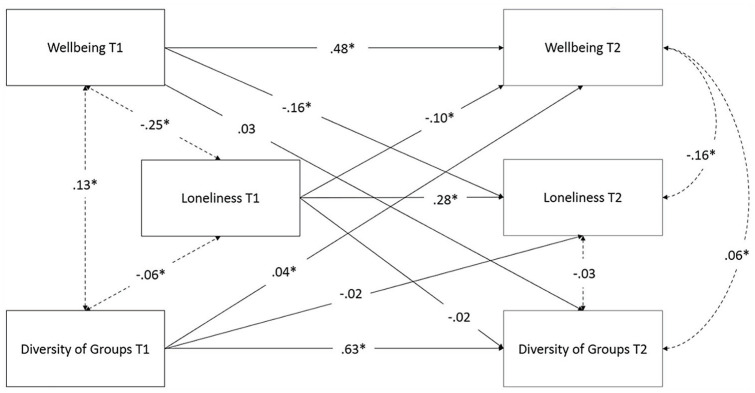
The Longitudinal Structural Equation Model, Assessing the Longitudinal Effects of Well-Being, Loneliness, and Diversity of Group Type on One Another. *Note.* After including social support as a covariate, all long-term predictive effects are significant except for Loneliness at time 1 to Diversity at time 2, Diversity at Time 1 to Loneliness at Time 2, and Wellbeing at time 1 to Diversity of Group Type at Time 2. Intra-timepoint relationships are shown in dotted lines, while inter-timepoint relationships are in complete lines. The standardized estimates for each effect is shown. Effects marked with an asterisk (*) were significant after inclusion of social support as a covariate.

A structural equation model (SEM) was constructed to assess the longitudinal relationship between group-type diversity and well-being while accounting for loneliness. The values of wellbeing and loneliness at T1 were included in the model to ensure that the within-participant variation in wellbeing is accounted for when assessing the long-term effects of group-type diversity on wellbeing. Figure 4 shows the results of the SEM. The descriptive statistics for each of the variables of interest are listed in Table 3, with Pearson’s correlations between the measures in Table 4.

**Table 3.** Descriptive Statistics for Study 2 Variables.

Variables	*N*	*M*	*SD*	Range
T1				
Group-Type Diversity	5,838	1.99	1.30	1–8
Well-being	5,838	1.83	0.23	1–2
Loneliness	5,838	2.35	1.85	1–7
T2				
Group-Type Diversity	5,838	1.81	1.14	1–8
Wellbeing	5,838	1.83	0.23	1–2
Loneliness	5,838	2.42	1.85	1–7

**Table 4.** Correlations Between Variables in Study 2.

Variables	1	2	3	4	5
1. T1_Group-Type Diversity					
2. T1_Wellbeing	.127				
3. T1_Loneliness	−.064	−.247			
4. T2_Group-Type Diversity	***.631***	.113	−.065		
5. T2_Well-being	.105	***.507***	−.221	.122	
6. T2_Loneliness	−.056	−.227	***.316***	−.070	−.268

*Note.* Bold and italics have been used to highlight the correlation between T1 and T2 of the same measure.

As expected, based on the findings from Study 1, T1 group-type diversity was a significant predictor of T2 wellbeing, controlling for both well-being and loneliness at T1, as well as for the relationship between group-type diversity and loneliness with wellbeing at T2 (β = .04, *Z* = 3.38, *p* = .001). Consistent with Study 1, T1 group-type diversity did not predict T2 loneliness (β = -.02, *Z* = -1.48, *p* = .139), nor was T1 loneliness a predictor of T2 group-type diversity (β = -.02, *Z* = -1.72, *p* = .086). All results, except for the link between wellbeing at time 1 and diversity of groups at time 2, remained significant after controlling for social support, suggesting that the level of social support offered by group membership is not the mechanism underlying the relationship between diversity of group type and wellbeing.

## Study 2 Discussion

Study 2 showed that T1 group-type diversity positively predicted T2 wellbeing, even when accounting for wellbeing and loneliness at T1. This supports and extends Study 1 through the use of longitudinal and country-wide data from a much larger sample than that used in Study 1. Although we did not test for the mediation of creative self-efficacy beliefs, based on the findings from Study 1, we recommend future research to test for longitudinal mediation pathways, including this important construct. In particular, in line with the assumptions of the SIAH (C. Haslam et al., 2018; Jetten, Haslam, & Haslam, 2012), we propose the idea that multiple and diverse social groups’ memberships substantially contribute to define our perception of who we are and provide individuals with a distinctive sense of “us” and “we.” What the present research adds to this well-established and empirically grounded approach is some understanding of the socio-cognitive mechanisms implied in this process, with individuals’ coping strategies and responses to life challenges being influenced by their perceptions of being able to flexibly reappraise the situation and creatively generate solutions. In other words, the meaning, purpose, and sense of belonging derived from diverse group memberships could furnish individuals’ identities with a robust sociocognitive scaffolding that enables them to reframe challenging situations. Flexible thinking and creative problem-solving could therefore be key in reframing negative self- and other perceptions, as in the case of loneliness: Rather than drawing exclusively upon dispositions and personal resources, individuals can obtain and nurture important beliefs through diverse group memberships which, in turn, will allow them to reframe their sense of being isolated and hopelessness and adapt their coping responses accordingly.

## General Discussion

The SIAH highlights that group identity is an important predictor of well-being (S. A. Haslam et al., 2005; Jetten, Haslam, & Alexander, 2012; Jetten, Haslam, & Haslam, 2012). Specifically, much of this work has suggested that having *multiple* group identities predicts better well-being outcomes (C. Haslam et al., 2018; Jetten et al., 2015; Lam et al., 2018; Steffens, Jetten, et al., 2016). We felt that the rationales laid out in previous literature—that membership of multiple groups is more likely to fulfill the needs of the individual, which leads to improved wellbeing—could be extended. In a pre-registered pilot study, we hypothesized that being a part of a larger number of different group-types would predict (a) lower levels of loneliness and (b) greater levels of well-being. We found that the positive relationship between group-type diversity and wellbeing was significant. The relationship’s effect size met our pre-specified smallest effect size of interest, so follow-up mediation models were conducted. These showed that creative self-efficacy and loneliness fully serially mediated the relationship between group-type diversity and wellbeing.

Mindful of the limits of cross-sectional and small-sample analyses, in Study 2, we conducted a longitudinal analysis on a secondary dataset that contained information on group type diversity, well-being, and loneliness. Longitudinal SEM analysis showed that diversity of group membership at T1 significantly predicted the well-being of participants at T2 (4 years later), even when controlling for their loneliness and well-being at T1, as well as accounting for the relationship between group-type diversity, loneliness, and well-being at T2. These results provide evidence for the temporal direction of the relationship from group-type diversity to well-being.

While only exploratory, these results fit well with established relationships. First, we propose that group-type diversity constitutes a further order of group-level predictors of well-being (alongside single and multiple group identifications). In line with our pre-registered hypothesis, our measure of group-type diversity was found to predict well-being across both studies. Our Study 2 lends further confidence to the interpretation of the direction of this relationship (from group-type diversity to well-being rather than the reverse). We propose that further exploration of the specific contribution of single, multiple, and diversity of group types on well-being would build upon these provisional results, and that investigation of the potential benefits of specific combinations of different group types would also be fruitful.

Second, our findings tie the literature on group-level predictors of creative self-efficacy to the literature on the health benefits of creative self-efficacy. While S. A. Haslam et al. (2013) and Steffens, Gocłowska, et al. (2016) have considered the benefits of identity processes for creativity, they did so separately from a consideration of the possible health benefits resulting from this effect. Conversely, researchers such as Fino and Sun (2022) have previously found creative self-efficacy to predict well-being (Fino & Sun, 2022; Tamannaeifar & Motaghedifard, 2014) but have not considered the group-level antecedents of this psychological attribute. Our research, then, adds to this broader research landscape by specifying an additional pathway via which group memberships can benefit health and simultaneously enriches the study of the health benefits of creativity by highlighting a way in which creative self-efficacy can be enhanced.

**Figure 5.**
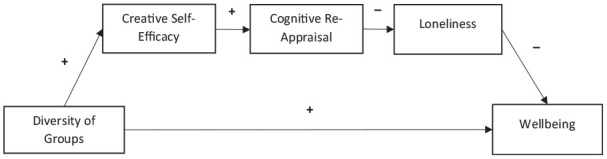
Proposed Theoretical Model for How Being a Diversity of Group Membership May Lead to Increased Well-Being. *Note.* Arrows marked with a + symbol indicate a theorized positive effect and arrows marked with a—symbol indicate a theorized negative effect. Group diversity is theorized to lead to increased wellbeing by increasing creative self-efficacy, which in-turn leads to a greater ability to re-appraise, and lessen, feelings of loneliness. Lower levels of loneliness would then lead to greater levels of wellbeing.

Third, we introduce a further explanatory factor in the pathway between group-type diversity and well-being. While the loneliness-reducing qualities of group identification have been documented extensively in the SIAH (e.g., McNamara et al., 2021), the relationship between group-related creativity and loneliness has not. To our knowledge, ours is the first study to find that the increased creative self-efficacy associated with group-type diversity is associated with a reduction in loneliness. However, we would suggest that this does accord with an understanding of both creative self-efficacy and loneliness: Loneliness is typically characterized by a cognitive bias toward attending to negative social stimuli and social threats (e.g., Cacioppo & Hawkley, 2009). Indeed, successful loneliness-reducing interventions typically employ a cognitive reframing element to allow reappraisal of one’s social situation (Dingle, Sharman, et al., 2021). We theorize that being a member of diverse group-types leads to increases in creative self-efficacy, which is associated with cognitive flexibility (Steffens et al., 2016). We propose that enhanced cognitive flexibility enables individuals to better regulate their cognitive assessment of loneliness, making them feel less lonely (see Figure 5 for the proposed model).

### Practical Implications

The implications of our findings for the reduction of loneliness and improvement of wellbeing are multiple. First, our findings suggest that group-based loneliness reduction interventions should involve consideration of the range of group-types to which their participants already belong. While increasing the number of group memberships may not decrease an individual’s loneliness per se (unless all of these groups have loneliness-reducing qualities), diversifying group-type memberships may have additional loneliness-reducing properties and benefits for well-being more generally. Second, while creativity has long been valued by cultural and artistic approaches to health and well-being, its precise mechanisms have remained largely undiscovered. Our findings in relation to the loneliness-reducing quality of creativity may provide grounds for the refinement and targeting of these types of approaches as part of community-based initiatives. Specifically, we would argue that forms of “Social Prescribing” which involve supporting patients to join and attend cultural and artistic groups (Stickley & Eades, 2013) would benefit from greater attention to how creativity may address the cognitive challenges posed by loneliness.

### Limitations

Study 1, although pre-registered, was only a pilot study. Consequently, its analyses were exploratory. As such, interpretations of these results, and of the theoretical models they imply, should be made with the exploratory nature of the analyses in mind. Study 2 helped corroborate the relationship between group-type diversity and well-being in a larger, more diverse sample, as well as demonstrating the temporal direction of the relationship. However, due to the nature of the dataset, the role of creative self-efficacy could not be assessed. Future studies should, therefore, involve conducting confirmatory research to examine if the results found in our two studies can be replicated with the specific aim of assessing the hypothesized path between group-type diversity and wellbeing (i.e., the model presented in Figure 5).

Another limitation of the current studies is that the measure of group-type diversity may not be distinguishable from the construct of multiple group membership that has already been shown to predict well-being benefits in prior literature (e.g., C. Haslam et al., 2018). The reason for this is, especially in Study 1, few individuals reported being members of more than one group-type (i.e., sport, religious, social, etc.). Future research would thus need to provide more concrete methods of distinguishing between the effects of being part of multiple groups (of the same type) and being part of a diverse range of group types (see Bentley et al., 2020).

## Conclusion

Past research has found that being a member of multiple groups has benefits for well-being outcomes but has not accounted for the effect of the group-type diversity. Remedying this, we found that group-type diversity predicts benefits in well-being via the serial mediators of increased creative self-efficacy and decreased loneliness. This contributes a new theoretical lens that SIAH researchers can use to assess the relationship between group membership and well-being. Practically, our findings suggest that health interventions that aim to improve wellbeing through group-based activities should consider the potential benefits of extending the range of their offer. As well as providing a greater range of social and psychological resources, increasing the diversity of patients’ group membership types may help unlock additional social and psychological benefits through increasing their creative potential.

## Supplemental Material

sj-docx-1-psp-10.1177_01461672231202278 – Supplemental material for Diversity of Group Memberships Predicts Well-Being: Cross-Sectional and Longitudinal Evidence Supplemental material, sj-docx-1-psp-10.1177_01461672231202278 for Diversity of Group Memberships Predicts Well-Being: Cross-Sectional and Longitudinal Evidence by Sarah J. Charles, Clifford Stevenson, Juliet R. H. Wakefield and Emanuele Fino in Personality and Social Psychology Bulletin

## Notes

1. In our pre-registration, we had planned to use the Benjamini-Yekutieli (2001) method to correct for multiple tests. However, based on guidance from peer-reviewers and having conducted further reading of the literature (e.g., Armstrong, 2014; Rubin, 2021) we are no longer alpha-correcting as the hypotheses are individual tests and not disjunction tests (Rubin, 2021).
