# Supplementary material for: Diversity of Group Memberships Predicts Well-Being: Cross-Sectional and Longitudinal Evidence
Source: Pers Soc Psychol Bull. 2023 Sep 30;51(5):716–29. doi: 10.1177/01461672231202278 (PMC11930635; doi:10.1177/01461672231202278)
Supplement: sj-docx-1-psp-10.1177_01461672231202278 – Supplemental material for Diversity of Group Memberships Predicts Well-Being: Cross-Sectional and Longitudinal Evidence [file sj-docx-1-psp-10.1177_01461672231202278.docx]

The Role of Group Type Diversity on Wellbeing – Supplementary Material

# Supplement to the method of Study 1

## Power Analysis

In the main manuscript of the article, for study 1, we provide two sensitivity power analyses for our two main hypotheses. The rationale for the parameters used in the power analysis (including the alpha value we consider significant) for study 1 is given below.

Using the pwr package (Champely et al., 2020) in R, a sensitivity power analysis for a linear relationship can be conducted using the pwr.r.test() function. Following the suggestions made by Bartlett and Charles (2021), we feel that justifying all of the parameters used is important. As this pilot study is the first study to assess the role of group type diversity on wellbeing and loneliness, we are not going to be overly-cautious to avoid Type I errors (false positive) as such an error can be better detected in a larger-scale, follow-up study. As such, we believe that setting our alpha (significance cut-off) value to 0.1 is fair. This means we are willing to accept that if the results of the analyses we conduct for our two main hypotheses are considered significant, this has a 10% likelihood of being a false positive. Using the same rationale – that this is a pilot study, and errors can be better detected upon replication – we are happy with using a power of 80%. Given the theory outlined in the Introduction section, our hypotheses were both directional: that *greater* group diversity would lead to *lower* loneliness (a directional, negative effect) or *higher* measure of general wellbeing (a directional positive effect). For this reason, the sensitivity power analyses used one-tailed tests.

## A full list of the measures contained in the questionnaire

As mentioned in the main manuscript. The survey used to collect the data of interest was part of a larger/wider project. As such, the questionnaire included measures that were not relevant to the current article. These include:

### ***Community Support***

An abridged (three-item) version of the Social Support Scale (Haslam et al., 2005) was used to measure social support. The original is a 10-item measure. The three items were:

1. Do you get the emotional support you need from other people in your neighbourhood?
2. Do you get the help you need from other people in your neighbourhood?
3. Do you get the advice you need from other people in your neighbourhood?

Responses were made on a five-point Likert scale, with the anchors Not at all/A little/Somewhat/A lot/Completely. The internal reliability of this measure was *α* = .89, *ω* = .89.

### ***Collective Self-Efficacy***

The Collective Efficacy Scale used by Reicher and Haslam in their study of prisons in the UK (Reicher & Haslam, 2006) was used. This is a five-item scale. The original article does not provide measures of internal reliability. In the original form, all of the items were prefaced with “my prison group…” or “when my prison group…”. We modified this to “My neighbourhood…” and “when my neighbourhood”. No other alterations were made. The items were as follows:

1. My neighbourhood is confident that we could deal with unexpected events
2. My neighbourhood can remain calm when facing difficulties because we can rely on our coping abilities
3. My neighbourhood can always manage to solve difficult problems if we try hard enough
4. When my neighbourhood is confronted with a problem, we can usually find several solutions
5. My neighbourhood can usually handle whatever comes our way

In line with the Reicher and Haslam (2006) usage, we used a five-point Likert scale response with the anchors Strongly Disagree/Disagree/Neither Agree nor Disagree/Agree/Strongly Agree. This measure had an internal reliability of *α* = .93, *ω* = .94

### ***Creative Self-Efficacy***

To measure creative self-efficacy, we used the three-item Creative Self-Efficacy Instrument (Tierney & Farmer, 2002, 2011) Each item (e.g., “I have confidence in my ability to solve problems creatively”) was rated on a 1-7 scale (strongly disagree-strongly agree). The Creative Self-Efficacy Instrument (Tierney & Farmer, 2002, 2011) is a three-item scale that uses either a seven-point Likert scale response (Tierney & Farmer, 2002) or a five-point Likert scale response (Tierney & Farmer, 2011). Given the choice between two scale lengths, the seven-point scale was chosen because a seven-point Likert scale leads to a faster completion time than for scales with more points (Matell & Jacoby, 1972), while also leading to a lower proportion of mid-point responses than a five-point Likert scale (Matell & Jacoby, 1972). This allows for the detection of greater levels of nuance. It has also been demonstrated that there is no significant psychometric difference between a six- and seven-point length scale (Simms et al., 2019), and that shorter than six items have lower short-term retest validity, while scales longer than seven items are not significantly more useful (Simms et al., 2019).

The three items were:

1. I have confidence in my ability to solve problems creatively
2. I fell that I am good at generating novel ideas
3. I have a knack for further developing the ideas of others.

Each of the seven points on the Likert scale were anchored with a verbal statement: Strongly disagree/Disagree/Slightly disagree/Neither agree nor disagree/Slightly agree/Agree/Strongly agree. The Creative Self-Efficacy Instrument has been shown to have moderate-to-high levels of internal reliability (α ranging from 0.74 to 0.87 across study populations). In this study, This measure had an internal reliability of *α* = .86, *ω* = .86.

### ***Neighbourhood Identification***

The single-item measure of social identification (Postmes et al., 2013) was used to assess the level to which participants identified with their local area on four levels (from most local, to widest coverage). The question is phrased as “I identify with other residents…”:

1. In my neighbourhood
2. In my local town
3. In Ashfield
4. In Nottinghamshire

Participants responded to each prompt using a seven-point Likert scale with the following anchors: Strongly disagree/Disagree/Slightly disagree/Neither agree nor disagree/Slightly agree/Agree/Strongly Agree. As it is a single-item measure, no measure of internal reliability is needed, however original article demonstrates the measure has good psychometric properties, including adequate test-retest reliability (see Postmes et al., 2013, p. 610)

### ***Community Belonging***

Participants were asked “Would you say that you feel a sense of belonging to your neighbourhood?”. The responses were given on a four-point Likert scale with verbal anchors of Definitely not / Probably not / Yes, probably / Yes, definitely.

### ***Community Trust***

Participants were asked “To what extent do you trust/distrust members of your neighbourhood?”. The responses were given on a five-point Likert scale with verbal anchors of Distrust/Slightly distrust/Neither trust nor distrust/Slightly Trust/Trust.

### ***Number of Groups***

Participants were also asked to provide exact numbers for each of the nine types of groups using the phrasing “How many [GROUP] groups do you belong to?”. Participants answered this using an open-text format, in case they chose to elaborate.

### ***Neighbourhood Needs***

Participants were asked how much they agreed with five statements about their neighbourhood about how they felt about the liveability of their local area. Responses to the questions were given on five-point Likert scales with the verbal anchors Strongly Disagree/Disagree/Neither Agree nor Disagree/Agree/Strongly Agree. The statements were: Living in my neighbourhood…

1. …makes me feel good about myself
2. …makes me feel as if I can achieve things I want to achieve
3. …gives me a sense of meaning of purpose
4. …makes me feel close to, or accepted by other people
5. …makes me feel supported by other people.

This was a measure constructed from a combination of measures that have been expressed in the literature, including that of Greenaway and colleagues (2016) who extended work by Williams (e.g., Williams, 2009) in suggesting that there are at least four core needs that group identity can fulfil that may play a role in wellbeing: *self-esteem* boosts that the group provides, the group allows for *control* of one’s fate (i.e., personal self-efficacy), the group provides *meaning*, as well as a sense of *belonging*. The fifth item, *support*, comes from the work of Genevieve Dingle, which suggests that support of the group is also important to wellbeing (Dingle et al., 2015; Dingle et al., 2021; Dingle & Sharman, 2022). As this was a newly constructed scale, an exploratory factor analysis was conducted. A scree plot suggested that a single-factor solution would be was appropriate, and a Horn’s parallel analysis (Glorfeld, 1995; Horn, 1965) also suggests that a single component is likely appropriate. Using an oblique rotation and a principal-axis method for extraction, this yielded a 1-factor solution that accounted for 66.73% of the variance. An oblique rotation was used because, in social sciences, different behaviours are rarely entirely uncorrelated. So, oblique rotations are more likely to lead to more useful solutions (Osborne, 2015). The internal reliability for this scale was *α* = .81, *ω* = .84.

***Barriers to Group Access***

The third version of the Barriers to Access to Care Evaluation scale (BACE-3; Clement et al., 2012) was used as a basis to create a seven-item measure to assess non-stigma-related barriers to access to engaging with local activities/groups. The list of items (below) were preceded by the question “Have any of the following ever stopped, delayed, or discoursed you from engaging with, or continuing to engage with, activities/groups in your local area?”

1. Problems with transportation or travel to activities or groups
2. Not being able to afford the financial cost of engaging with the activities or groups
3. Difficulty fitting the activities or groups around work or other commitments
4. Unavailability of activities or groups that appeal to me personally
5. Unavailability of activities or groups that suit my own ethnic or cultural group
6. Feelings of anxiety about attending groups
7. A negative experience with an activity or group

Responses were given on a four-item scale, to conform with the BACE-3 scale (Clement et al., 2012), with the anchors: not at all / a little / quite a lot / a lot. A scree plot makes it unclear how many components/factors that this scale fits onto, ranging from 1 to 3. However, a Horn’s parallel analysis (Glorfeld, 1995; Horn, 1965) suggests 1 factor is likely appropriate. Following this parallel analysis, a the items were loaded onto a single factor. The internal reliability for this scale was *α* = .64, *ω* = .75. This suggests low-to-moderate reliability. The discrepancy between *α* and *ω* suggests that at least one of the assumptions of *α*: unidimensionality, tau-equivalence, or uncorrelated errors, may have been violated, causing it to mis-estimate the reliability. As the factor structure was ambiguous, it is likely that treating this as a unidimensional measure is not appropriate.

### ***Financial Managing***

Participants were asked the question “How well would you say you yourself are managing financially these days?” Responses were given on a five-item scale with the verbal anchors Very difficult / Quite difficult / Just about getting by / Doing alright / Living comfortably. This question has been used in the British Household Panel Survey (Taylor & Brice, 1998).

### ***Loneliness***

of the time/never). The UCLA-3 (Hughes et al., 2004) is a three-item, shortened variant of the 20-item UCLA Loneliness Scale (Russell, 1996). The three items are as follows:

1. How often do you lack companionship?
2. How often do you feel left out?
3. How often do you feel isolated from others?

The three-item version uses the same three-point Likert scale as the original UCLA Loneliness Scale, with verbal anchors being Hardly Ever / Some of the Time / Often. For this study, The internal reliability was *α* = .88, *ω* = .89.

### ***Wellbeing***

The five-item World Health Organisation Well-Being Index (WHO-5; World Health Organization, 1998) was used as a measure of general wellbeing. The measure asks participants to rate how well the five statements (e.g., “I have felt cheerful and in good spirits”) relate to them *over the last 14 days* using a 0-5 scale (at no time-all of the time). As per instructions, participants’ scores were summed then multiplied by 4 to create a 0-100 scale.

1. I have felt cheerful and in good spirits
2. I have felt calm and relaxed
3. I have felt active and vigorous
4. I woke up feeling fresh and rested
5. My daily life has been filled with things that interest me

The responses are to be given on a six-point Likert scale with verbal anchors: At no time/Some of the time/Less than half of the time/More than half of the time/Most of the time/All of the time. Scores are averaged on the measure. A higher score means the participant has better general wellbeing.

0A systematic review of the WHO-5 measure has found it to have high levels of clinometric validity, with high responsiveness and sensitivity in controlled clinical trials as well as being a valid screening tool for depression (Topp et al., 2015). For this study, the internal reliability was *α* = .90, *ω* = .93.

### ***Number of Doctor Appointments***

The number of doctor/GP appointments that the participant has had in the last 3 months was also measured, with participants providing a number (0-11), or choosing “12 or more”.

### ***Demographic Variables***

Information about a participants’ age, gender, ethnicity, relationship status, highest level of qualification, sexual orientation, disability status, and employment situation were also gathered.

## **Correction for Multiple Tests**

In our pre-registration, we had planned to use the Benjamini-Yekutieli (Benjamini & Yekutieli, 2001; Yekutieli & Benjamini, 1999) method to correct for multiple tests. However, based on guidance from peer-reviewers and having conducted further reading of the literature (see, Armstrong, 2014; Rubin, 2021), we are no longer alpha-correcting as the hypotheses are individual tests and not disjunction tests (Rubin, 2021).

## **Procedure**

29,835 survey invites were sent out via a physical mail-out conducted through the Royal Mail Door-to-Door service. All houses within selected post-code areas were sent the survey invite. The selected post-codes included all of those within the three major areas within Ashfield: Hucknall, Sutton-in-Ashfield, and Kirkby-in-Ashfield. The invites contained information about the survey, as well as a website address and QR code, both leading to the questionnaire website Qualtrics (Qualtrics, 2019). Upon loading the survey on Qualtrics, participants were shown the information sheet again, followed by a consent form. Once participants completed the consent form, Qualtrics then allows them to complete the remainder of the survey. After completing the survey, Qualtrics then displays a debrief page providing further information about who to contact regarding the study, as well as contact information for the mental health charity MIND, should the participant feel they need to contact them.

Finally, participants were given the option to indicate whether they would be interested in taking part in a follow-up survey (beyond the scope of the current pre-registration), as well as the choice to enter a prize draw for vouchers worth £400.

# Supplement to Method of Study 2

## Power Analysis

Pan et al. (2018) produced a series of simulations for longitudinal structural equational models. In their study they provided the approximate number of participants that would be needed given various effect sizes, intra-class correlations, and using different estimation methods (In Tables 1-5 in their article). Pan et al. (2018) found that samples with higher ICC values would require greater participant numbers. However, the largest sample size they found was necessary using the most conservative estimation model (Sobel) was 638 participants in the case of assuming small effect sizes (β = 0.14), with very high ICC values (0.9), with only two time points.

The benefit of having secondary data is that we can calculate the ICC for out outcome variable of interest from the data ahead of time. The ICC of Wellbeing (the outcome measure of interest) is 0.51 (95%CI: 0.49 – 0.53). The closest Pan et al. (2018) come to this is providing a table for ICC = 0.6 (Table 4), which shows that even assuming their smallest effect size, with two time points, and their most conservative estimation model, a sample size of 551 would be appropriate for a longitudinal mediation model. The sample we have in the ELSA is an order of magnitude larger, which suggests this analysis is more than appropriately powered.

# Supplement to the Results

## Note about significance

### ***Study 1***

As Study 1 was a pilot study, with a fairly small sample size (N = 328), and with a large number of analyses to be conducted, alpha was set to 0.1, after correcting for multiple testing. This means that we would accept any BY-corrected *p*-values below 0.1 as statistically significant. We have marked significant results, even after BY-correction with an asterisk (*).

### ***Study 2***

As study 2 was a follow-up study, with a much larger sample size (N = 5,838), and specified relationships based on the results of study 1, we wanted to be more conservative in whether we accepted results as statistically significant, and so a more conservative alpha of 0.01 was used, after correcting for multiple tests. As with the tables presented in Study 1, Results that remained significant at the stated alpha level after correcting for multiple tests are marked with an asterisk (*)

# Study 1 Full Model Outputs

A note about the models we created:

While we understand that further model creation was possible with sound theoretical reasoning, we did not wish to continue building more models, as this would cause a greater risk of Type II errors from occurring due to correcting for the fact that we are running multiple tests.

## Model 1: Creativity as a Mediator

|  | | | | |  |
| --- | --- | --- | --- | --- | --- |
| **Variables** | **Estimate** | **Std Err** | **Z** | ***p*-value** |  |
| **Predicting WHO-5 (Wellbeing)** | | | | |  |
| Group Type Diversity (Direct) | 1.85 | 1.71 | 1.06 | .289 |  |
| Creative Self-Efficacy* | 4.17 | 1.01 | 4.14 | <.001 |  |
| **Predicting Creative Self-Efficacy** | | | | |  |
| Group Type Diversity* | 0.27 | 0.94 | 2.91 | .004 |  |
| **Residual Variances** | | | | |  |
| WHO5 (Wellbeing) | 513.55 | 40.55 | 12.67 | <.001 |  |
| Creative Self-Efficacy | 1.58 | 0.13 | 12.67 | <.001 |  |
| **Constructed Variables** | | | | |  |
| Indirect* | 1.14 | 0.48 | 2.38 | .017 |  |
| Total* | 2.95 | 1.73 | 1.70 | .089 |  |


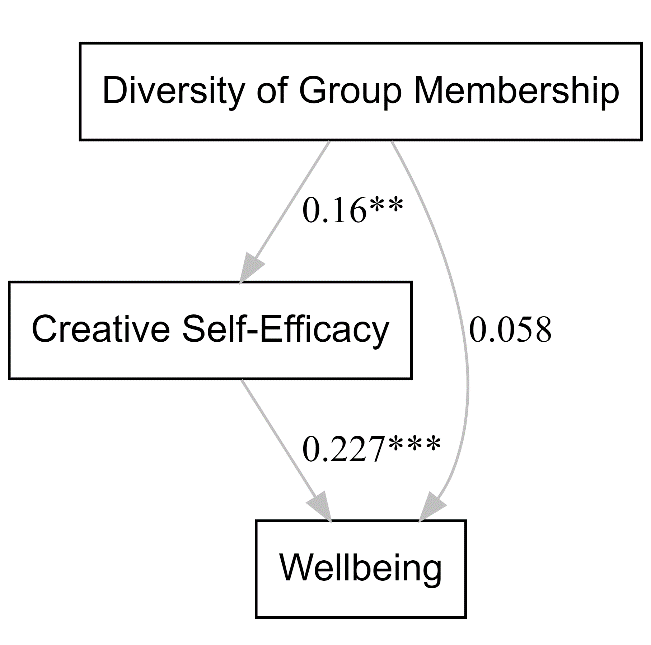


***Supplemental Figure 1***. The output of the first exploratory model (a), with creative self-efficacy as the only mediator. The significant mediation was maintained even when accounting for Social Support as a covariate (b).


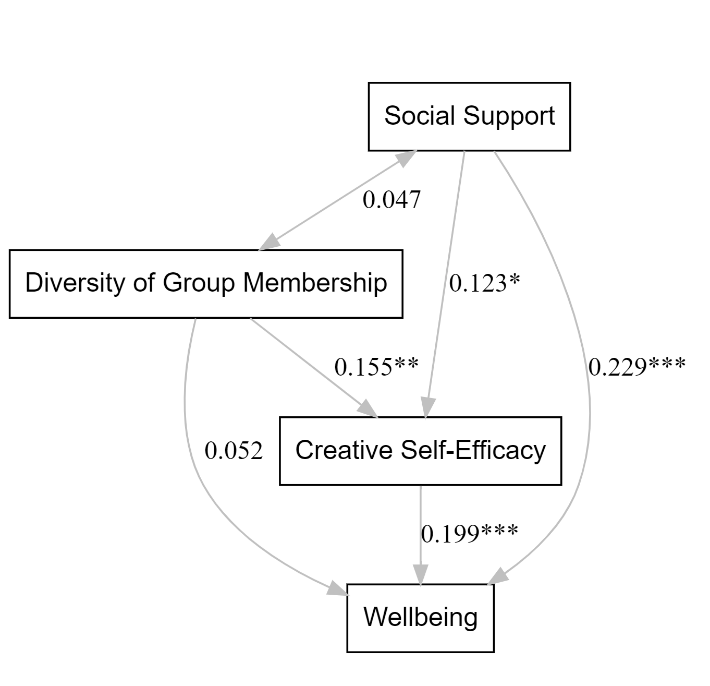


***(a)***

***(b)***

## Model 2: Creativity and Loneliness as Serial Mediators

| ***Supplemental Table 2.*** Table showing the output from the lavaan model for the second exploratory mediation analysis shown in the main manuscript: the serial mediation of creative self-efficacy then loneliness on the effect of group type diversity on wellbeing | | | | | |
| --- | --- | --- | --- | --- | --- |
| **Variables** | **Estimate** | **Std. Err.** | **Z** | ***p-value*** | |
| **Predicting WHO5 (Wellbeing)** | | | | | |
| Loneliness (c)* | -19.32 | 1.65 | -11.67 | <.001 |  |
| Creative Self-Efficacy (e)* | 2.19 | 0.86 | 2.55 | .011 |  |
| Group Type Diversity (f; Direct) | 1.65 | 1.43 | 1.15 | .251 |  |
| **Predicting Creative Self-Efficacy** | | | | | |
| Group Type Diversity (a)* | 0.27 | 0.09 | 2.91 | .004 |  |
| **Predicting Loneliness** | | | | | |
| Creative Self-Efficacy (b)* | -0.10 | 0.03 | -3.60 | <.001 |  |
| Group Type Diversity (d) | -0.01 | 0.05 | -0.18 | .885 |  |
| **Residual Variances** | | | | | |
| WHO5 (Wellbeing) | 360.52 | 28.46 | 12.67 | <.001 |  |
| Creative Self-Efficacy | 1.58 | 0.13 | 12.67 | <.001 |  |
| Loneliness | 0.41 | 0.03 | 12.67 | <.001 |  |
| **Constructed Variables** | | | | | |
| Serial Indirect (abc)* | 0.54 | 0.24 | 2.22 | .026 |  |
| Indirect 2 (ae) | 0.60 | 0.31 | 1.91 | .055 |  |
| Indirect 3 (dc) | 0.17 | 0.93 | 0.18 | .856 |  |
| Indirect 4 (bc)* | 1.98 | 0.58 | 3.44 | .001 |  |
| Total Mediation (abc+ae+dc+f)* | 2.95 | 1.73 | 1.70 | .089 |  |
| Total Non-direct (bc+e)* | 4.17 | 1.01 | 4.14 | <.001 |  |


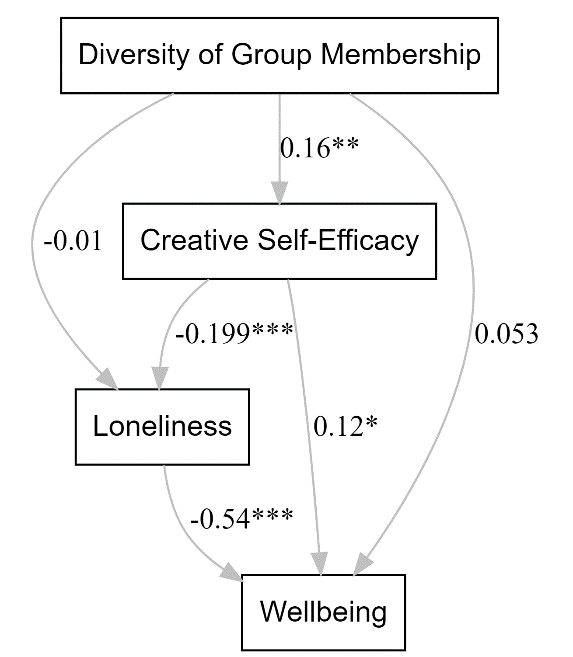

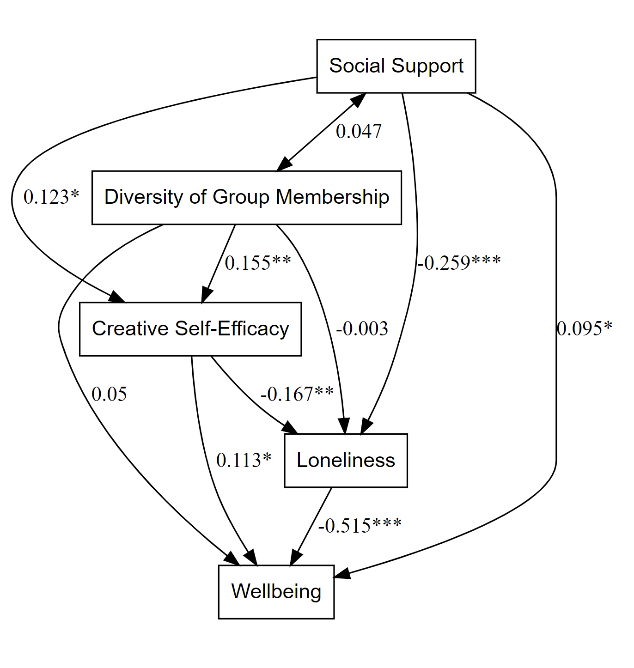


***Supplemental Figure 2***. The output of the second exploratory model, (a) with the serial mediation of creative self-efficacy and loneliness. The significant mediation was maintained even when accounting for Social Support as a covariate (b).

***(b)***

***(a)***

## Model 3: Collective Self-Efficacy as a Mediator

| ***Supplemental Table 3.***  Table showing the output from the lavaan model for the third exploratory mediation analysis shown in the main manuscript: the mediation of Community self-efficacy on the effect of group type diversity on wellbeing | | | | |  |
| --- | --- | --- | --- | --- | --- |
| **Variables** | **Estimate** | **Std Err** | **Z** | ***p*-value** |  |
| **Predicting WHO-5 (Wellbeing)** | | | | |  |
| Group Type Diversity (Direct)* | 2.75 | 1.70 | 1.62 | .105 |  |
| Community Self-Efficacy* | 6.37 | 1.67 | 3.81 | <.001 |  |
| **Predicting Collective Self-Efficacy** | | | | |  |
| Group Type Diversity | 0.03 | 0.06 | 0.55 | .582 |  |
| **Residual Variances** | | | | |  |
| WHO5 (Wellbeing) | 517.61 | 40.86 | 12.67 | <.001 |  |
| Community Self-Efficacy | 0.58 | 0.05 | 12.67 | <.001 |  |
| **Constructed Variables** | | | | |  |
| Indirect | 0.20 | 0.36 | 0.55 | .586 |  |
| Total* | 2.95 | 1.73 | 1.70 | .089 |  |


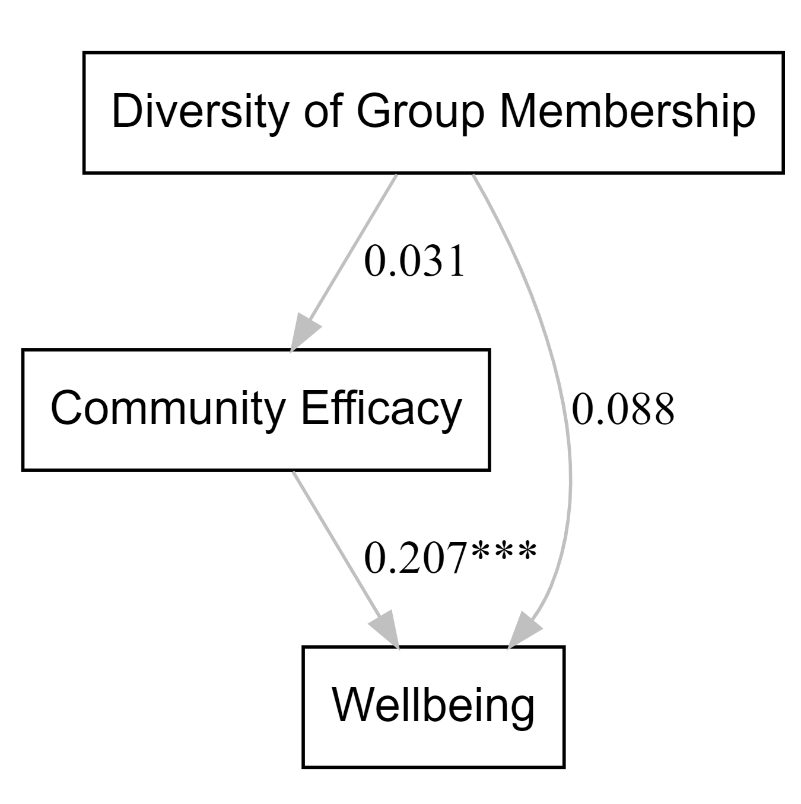


***Supplemental Figure 3***. The pre-registered mediation model (Häusser et al., 2020), where collective self-efficacy, aka community efficacy, was tested as a mediator.

# Study 2 Full Model Output

## Model Output

| ***Supplemental Table 4.***  Table showing the output from the lavaan model for the longitudinal analysis in the main manuscript for Study 2. | | | | |  |  |
| --- | --- | --- | --- | --- | --- | --- |
| **Variables** | **Estimate** | **Std. Err.** | **Z** | ***p*** |  |  |
| **Predicting Wellbeing at Time 2** | | | | |  |  |
| T1 Wellbeing * | 0.48 | 0.01 | 41.02 | <.001 | |  |
| T1 Loneliness * | -0.01 | 0.00 | -8.76 | <.001 | |  |
| T1 Group Type Diversity * | 0.01 | 0.00 | 3.38 | .001 | |  |
| **Predicting Loneliness at Time 2** | | | | |  |  |
| T1 Wellbeing * | -1.26 | 0.10 | -12.25 | <.001 | |  |
| T1 Loneliness * | 0.28 | 0.01 | 21.88 | <.001 | |  |
| T1 Group Type Diversity | -0.03 | 0.02 | -1.48 | .139 | |  |
| **Predicting Group Type Diversity at Time 2** | | | | | |  |
| T1 Wellbeing * ^a^ | 0.14 | 0.05 | 2.73 | .006 | |  |
| T1 Loneliness | -0.01 | 0.01 | -1.72 | .086 | |  |
| T1 Group Type Diversity * | 0.55 | 0.01 | 61.15 | <.001 | |  |
| **Residual Variances** | | | | |  |  |
| T1 Group Type Diversity | 1.70 | 0.03 | 54.02 | <.001 | |  |
| T1 Loneliness | 3.43 | 0.06 | 54.02 | <.001 | |  |
| T1 Wellbeing | 0.05 | 0.00 | 54.02 | <.001 | |  |
| T2 Group Type Diversity | 0.78 | 0.01 | 54.02 | <.001 | |  |
| T2 Loneliness | 3.00 | 0.06 | 54.02 | <.001 | |  |
| T2 Wellbeing | 0.04 | 0.00 | 54.02 | <.001 | |  |
| **Residual Covariances** | | | | |  |  |
| T1 Loneliness ~~ T1 Diversity | -0.16 | 0.03 | -4.90 | <.001 | |  |
| T1 Wellbeing ~~ T1 Diversity | 0.04 | 0.00 | 9.62 | <.001 | |  |
| T1 Wellbeing ~~ T1 Loneliness | -0.10 | 0.01 | -18.29 | <.001 | |  |
| T2 Loneliness ~~ T2 Diversity | -0.05 | 0.02 | -2.45 | .014 | |  |
| T2 Wellbeing ~~ T2 Diversity | 0.01 | 0.00 | 4.36 | <.001 | |  |
| T2 Wellbeing ~~ T2 Loneliness | -0.05 | 0.00 | -11.86 | <.001 | |  |
| ^a^ This was no longer significant in the model that including social support, see R file.  * significant at least to the 0.01 alpha level. | | | | | |  |

## Longitudinal Model Figure

As shown in Supplemental Figure 4, we see that, as expected after the results from study 1, Wellbeing at time 2 is significantly predicted by Group Type Diversity at time 1, but Loneliness at time 2 is not predicted by Group Type Diversity at time 1. Importantly, Wellbeing at time 1 does not significantly predict Group Type Diversity at time 2, which suggests a specific direction of effect: Group Type Diversity leads to improved wellbeing over time, but better wellbeing does not lead to being in more diverse types of groups.

***Supplemental Figure 4***. The output of the longitudinal model, shown in more clear detail in the main manuscript. The Time 2 variables are shown in **blue,** while the Time 1 variables are shown in **black**. The colour of the arrows shows whether or not they are significant. **Grey** is significant. **Purple** is not significant at the 0.01 level after inclusion of social support as a covariate, but would be significant at the 0.1 level that was used in study 1. **Red** is not significant even at the 0.1 level.


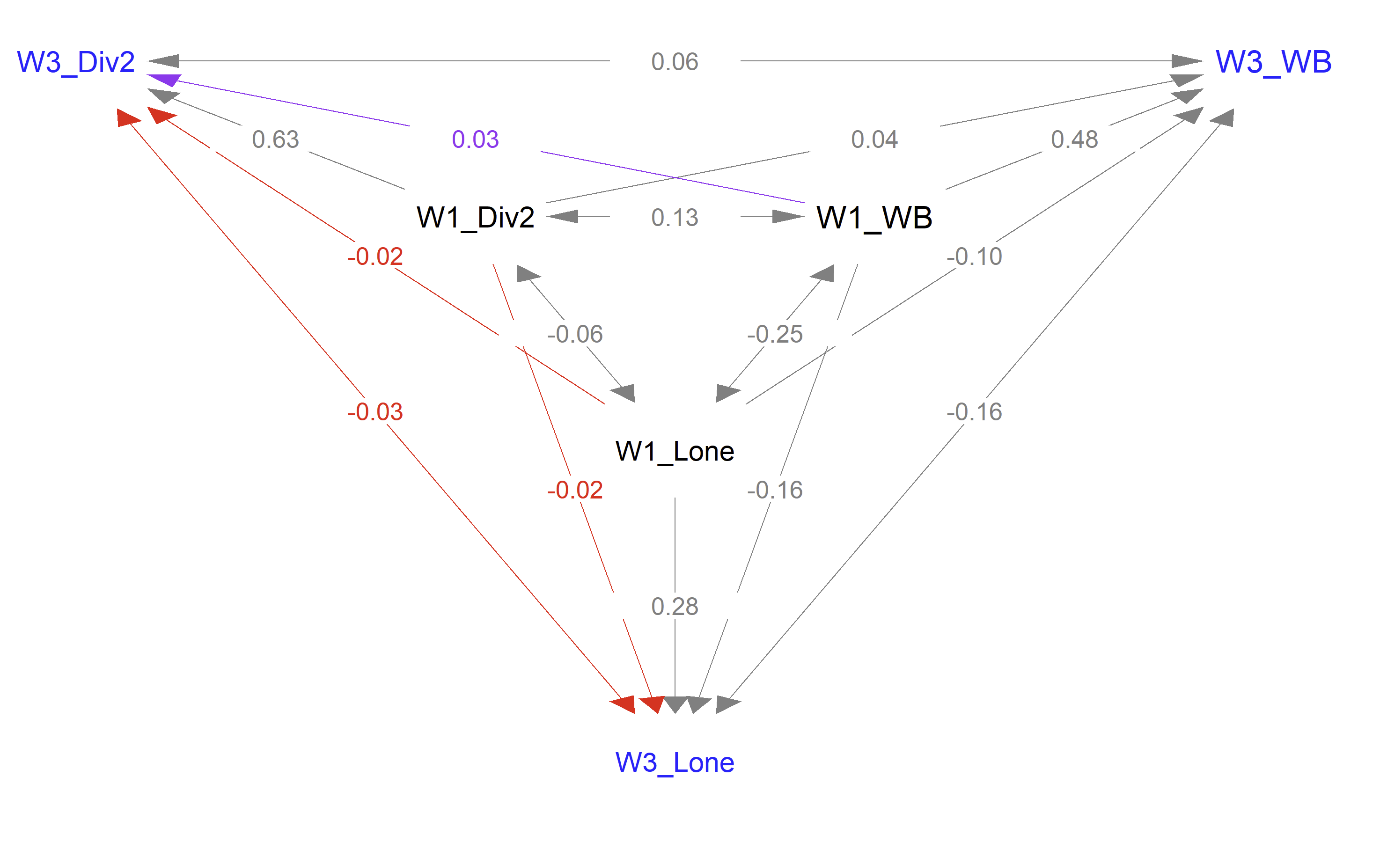


Including Social Support into the model showed that wellbeing at time 1 no longer predicted group type diversity at time 2, but all other results remained significant (all previously significant results remained *p* < 0.01):

# References

Armstrong, R. A. (2014). When to use the B onferroni correction. *Ophthalmic and Physiological Optics*, *34*(5), 502-508.

Bartlett, J. E., & Charles, S. (2021). Power to the People: A Beginner’s Tutorial to Power Analysis using jamovi.

Benjamini, Y., & Yekutieli, D. (2001). The control of the false discovery rate in multiple testing under dependency. *Annals of statistics*, 1165-1188.

Champely, S., Ekstrom, C., Dalgaard, P., Gill, J., Weibelzahl, S., Anandkumar, A., Ford, C., Volcic, R., De Rosario, H., & De Rosario, M. H. (2020). Package ‘pwr’. In *R package version* (Vol. 1).

Clement, S., Brohan, E., Jeffery, D., Henderson, C., Hatch, S. L., & Thornicroft, G. (2012). Development and psychometric properties the Barriers to Access to Care Evaluation scale (BACE) related to people with mental ill health. *BMC Psychiatry*, *12*(1), 1-11.

Dingle, G. A., Cruwys, T., & Frings, D. (2015). Social identities as pathways into and out of addiction. *Frontiers in Psychology*, *6*, 1795.

Dingle, G. A., Ingram, I., Haslam, C., & Kelly, P. J. (2021). Taking social identity into practice. In *The Handbook of Alcohol Use* (pp. 511-530). Elsevier.

Dingle, G. A., & Sharman, L. S. (2022). Social Prescribing: A Review of the Literature. *Existential Concerns and Cognitive-Behavioral Procedures*, 135-149.

Glorfeld, L. W. (1995). An improvement on Horn's parallel analysis methodology for selecting the correct number of factors to retain. *Educational and Psychological Measurement*, *55*(3), 377-393.

Greenaway, K. H., Cruwys, T., Haslam, S. A., & Jetten, J. (2016). Social identities promote well‐being because they satisfy global psychological needs. *European journal of social psychology*, *46*(3), 294-307.

Haslam, S. A., O'Brien, A., Jetten, J., Vormedal, K., & Penna, S. (2005). Taking the strain: Social identity, social support, and the experience of stress. *British Journal of Social Psychology*, *44*(3), 355-370.

Horn, J. L. (1965). A rationale and test for the number of factors in factor analysis. *Psychometrika*, *30*(2), 179-185.

Hughes, M. E., Waite, L. J., Hawkley, L. C., & Cacioppo, J. T. (2004). A short scale for measuring loneliness in large surveys: Results from two population-based studies. *Research on aging*, *26*(6), 655-672.

Matell, M. S., & Jacoby, J. (1972). Is there an optimal number of alternatives for Likert-scale items? Effects of testing time and scale properties. *Journal of Applied Psychology*, *56*(6), 506.

Osborne, J. W. (2015). What is rotating in exploratory factor analysis? *Practical Assessment, Research, and Evaluation*, *20*(1), 2.

Pan, H., Liu, S., Miao, D., & Yuan, Y. (2018). Sample size determination for mediation analysis of longitudinal data. *BMC medical research methodology*, *18*(1), 1-11.

Postmes, T., Haslam, S. A., & Jans, L. (2013). A single‐item measure of social identification: Reliability, validity, and utility. *British Journal of Social Psychology*, *52*(4), 597-617.

Qualtrics. (2019). *Qualtrics*. In (Version 2019) Qualtrics. <https://www.qualtrics.com>

Reicher, S., & Haslam, S. A. (2006). Rethinking the psychology of tyranny: The BBC prison study. *British Journal of Social Psychology*, *45*(1), 1-40.

Rubin, M. (2021). When to adjust alpha during multiple testing: A consideration of disjunction, conjunction, and individual testing. *Synthese*, *199*(3-4), 10969-11000.

Russell, D. W. (1996). UCLA Loneliness Scale (Version 3): Reliability, validity, and factor structure. *Journal of personality assessment*, *66*(1), 20-40.

Simms, L. J., Zelazny, K., Williams, T. F., & Bernstein, L. (2019). Does the number of response options matter? Psychometric perspectives using personality questionnaire data. *Psychological assessment*, *31*(4), 557.

Taylor, M. F., & Brice, J. (1998). British Household Panel Survey user manual: introduction, technical reports and appendices. *ESRC Research Centre On Micro-Social Change, University Of Essex, Colchester. URL:* [*http://www*](http://www)*. iser. essex. ac. uk/bhps*.

Tierney, P., & Farmer, S. M. (2002). Creative self-efficacy: Its potential antecedents and relationship to creative performance. *Academy of Management journal*, *45*(6), 1137-1148.

Tierney, P., & Farmer, S. M. (2011). Creative self-efficacy development and creative performance over time. *Journal of Applied Psychology*, *96*(2), 277.

Topp, C. W., Østergaard, S. D., Søndergaard, S., & Bech, P. (2015). The WHO-5 Well-Being Index: a systematic review of the literature. *Psychotherapy and Psychosomatics*, *84*(3), 167-176.

Williams, K. D. (2009). Ostracism: A temporal need‐threat model. *Advances in experimental social psychology*, *41*, 275-314.

World Health Organization. (1998). *Wellbeing measures in primary health care/the DepCare Project: report on a WHO meeting: Stockholm, Sweden, 12–13 February 1998*.

Yekutieli, D., & Benjamini, Y. (1999). Resampling-based false discovery rate controlling multiple test procedures for correlated test statistics. *Journal of Statistical Planning and Inference*, *82*(1-2), 171-196.
